# Streamlining and cycle time reduction of the startup phase of clinical trials

**DOI:** 10.1186/s13063-020-4079-8

**Published:** 2020-01-29

**Authors:** Amani Abu-Shaheen, Ahmad Al Badr, Isamme Al Fayyad, Adel Al Qutub, Eissa Ali Faqeih, Mohamad Al-Tannir

**Affiliations:** Research Center, King Fahad Medical City, P.O Box 59046, Riyadh, 11525 Saudi Arabia

**Keywords:** Clinical trials, Cycle time, Startup, Streamline, Ethics approval, Institutional review board, Investigational drug service, Pathology, Clinical laboratory medicine

## Abstract

**Objective:**

The startup phase of a clinical trial (CT) plays a vital role in the execution of new drug development. Hence, the aim of this study is to identify the factors responsible for delaying the CT startup phase. Further, it focuses on streamlining and reducing the cycle time of the startup phase of newly sponsored CTs.

**Methods:**

Thirteen sponsored CTs conducted between 2016 and 2017 at the Clinical Research Department of King Fahad Medical City, Riyadh, Saudi Arabia, were considered for this study. Eight trials were analyzed to identify the data specific to startup metrics using the FOCUS–PDCA cycle (**F**ind an improvement area–**O**rganize a team–**C**larify current practices–**U**nderstand the source of variation/problem–**S**elect a Strategy–**P**lan–**D**o–**C**heck–**A**ct). Six measures incorporated in the metrics were (1) date of initial contact with site to the signing of confidentiality agreement, (2) date of receiving questionnaire from sponsor to date of its completion, (4) time taken to review protocol and approve investigational drug service form, and (5) time taken to study protocol and approve pharmacy and pathology and clinical laboratory medicine form and date of receipt of institutional review board (IRB) submission package to final IRB approval. Fishbone analysis was used to understand the potential causes of process variation. Mean (SD) time was calculated for each metric before and after implementation of the intervention protocol to analyze and compare percentage reduction in the mean cycle time of CTs. Data were represented as mean (SD), and the *P* value was calculated for each metric. The significance level was set at *P* < 0.05.

**Results:**

Of the various potential factors of delay identified through fishbone analysis, the two major ones were lack of a well-defined timeline for approval and review of the study protocol and inconsistent IRB meetings. After introduction of the new intervention protocol, the entire CT life cycle was reduced by 45.6% (mean [SD], 24.8 [8.2] weeks vs. 13.5 [11.6] weeks before and after the intervention, respectively).

**Conclusion:**

Various factors are responsible for the delay of the startup phase of CTs, and understanding the impact of each element allows for optimization and faster execution of the startup phase of CTs.

## Introduction

Clinical trials (CTs) are essential for testing new drugs and devices and determining the effectiveness of various new therapeutic strategies and diagnostic tests [1, 2]. However, several challenges mask the success of a CT, such as diverse stakeholders (sponsors, regulators, investigators, physicians, payers, and patients), infrastructure, logistics, time, or other support systems (informatics and human resources) [3]. It has been noted that the startup phase of a trial (selecting and preparing trial sites for initiation) sets the tone and plays an essential role in determining the study’s success. However, initiating a CT is a complicated and time-consuming process. It requires a significant understanding of various ethical committees, regulatory bodies, and insights into several significant steps, such as protocol writing, funding application, and obtaining approval from involved stakeholders [4].

Additionally, difficulty in patient recruitment is considered one of the significant reasons for the delay in the overall drug development process [2]. The initiation of a new CT usually starts with a research hypothesis, followed by protocol writing; budget and contract negotiation; essential regulatory document collection; development of a patient recruitment strategy; and approval from ethical bodies, research and development (R&D) departments, and other regulatory bodies [4, 5]. Overall, the startup phase constitutes an administrative and logistical undertaking [6]. However, the duration of the startup phase generally varies among sites and depends on trial complexity [6, 7].

Lamberti et al. noted that early stages of study initiation cause the majority of the lag time (time taken from discovery or start of the research to its implementation in clinical practice) where variation in cycle time to the first patient occurs by site type (longest for academic institutions and government-funded sites and fastest for physician practices) [8, 9]. Krafcik et al. mentioned that 86% of CTs experience delays abiding by the startup timeline set by the sponsor and contract research organization, and with a site maintenance cost of up to $2500 per month, trial delays could cost the sponsors dearly [6]. On the basis of our experience at the research center in King Fahad Medical City (KFMC), Saudi Arabia, the cycle time of the startup phase of any new sponsored CT is unusually prolonged, owing to various factors that negatively impact trial conduct. Therefore, the aim of the current study was to identify the factors that may play a role in delaying the startup phase of CTs. Also, the study aimed to streamline and reduce the cycle time of the startup phase of new sponsored CTs.

## Materials and methods

This study consists of three parts, namely, preassessment, intervention, and reassessment. The study was conducted between February 2016 and 2017 at the Clinical Research Department of KFMC in Riyadh, Saudi Arabia. All newly conducted sponsored trials during the study period were eligible to participate in this study. No trials were excluded from the analysis.

In Saudi Arabia, the approval for CTs is given by local institutional review boards (IRBs), which are mainly located in hospitals. After getting IRB approval, one can apply for approval from the Saudi Food and Drug Authority.

A total of 13 CTs were included in this study, of which 8 trials were in the preassessment phase and 5 were in the postassessment phase. The trials were related to drug investigations, were multicentered, were in phase 3 and phase 4, and had similar requirements. The characteristics of the pre- and postintervention trials considered for our study are presented in Table 1.

**Table 1:** Preintervention and postintervention study characteristics

Study characteristics	Preintervention (*n* = 8) *n* (%)	Postintervention (*n* = 5) *n* (%)
Health field		
Cardiac and pulmonary	5 (62.5)	3 (60.0)
Cancer and hematology	3 (37.5)	2 (40.0)
Patient population		
Adult	6 (75.0)	4 (80.0)
Pediatric	2 (25.0)	1 (20.0)
Industry-sponsored study	8 (100)	5 (100)
Study intervention		
Drug	8 (100)	5 (100)
Phase		
III	5 (62.5)	3 (60.0)
IV	3 (37.5)	2 (40.0)
International studies/multicenter studies	8 (100)	5 (100)

In the preassessment phase (February–April 2016), eight CTs were analyzed to identify the accomplished data-specific startup metrics. We organized a team of four different disciplines, composed of individuals who were the key persons involved in the startup phase (date of the first contact with the sponsor to the date of getting the IRB approval), including a senior clinical research specialist and chairperson of the Clinical Research Department, chairpersons of the IRB, investigational drug service (IDS) pharmacy, and pathology and clinical laboratory medicine (PCLM) departments. The team was charged with the responsibility to assess the current situation and shorten the time needed for the startup phase of the newly sponsored CTs using the FOCUS-PDCA cycle (**F**ind an improvement area–**O**rganize a team–**C**larify current practices–**U**nderstand source of variation/problem–**S**elect a strategy Plan–Do–Check–Act) [10]. FOCUS-PDCA is an effective method for solving a simple/complex clinical process problem systematically. It aids in problem-solving, change implementation, and continuous improvement in the process [11]. We examined the current process for the startup phase of new sponsored clinical studies and used a fishbone analysis (a tool for categorizing potential causes of a problem to determine its root causes) to clarify the current knowledge of the process and to understand the causes of process variation [12].

We used the data available at our site from the previous startups to characterize the role of each element of a study with the time required to attain milestones during the startup phase. The metrics incorporated five measures: (1) the date of initial contact with the site to the date of actual signing of the confidentiality agreement, (2) the date of receiving the feasibility questionnaire from the sponsor to the date of its completion, (3) time taken by the IDS pharmacy to review the study protocol and approve the IDS form, (4) time taken by the PCLM to review the study protocol and approve the PCLM form, and (5) date of receipt of the IRB submission package by the site through the date of submission to the IRB and date of final IRB approval. The number of days to IRB approval was calculated from the time of receiving the IRB submission package from the sponsor to the time between IRB package submission to the final IRB approval.

The intervention started in May 2016. We implemented the following interventions to streamline and reduce the cycle time of the startup phase of five new sponsored CTs: (1) arranging meetings with IRB chairpersons, IDS pharmacy, and PCLM departments to agree on a specified timeline for approving further studies; (2) completing the IDS form within 7–10 working days; (3) completing the PCLM form within 5–10 working days; (4) modified industry-sponsored research committee for the protection of persons approval; (5) lab coordinators to handle all issues related to the send-out lab and get the prices for different tests; (6) meeting with IRB chairperson, IRB members, and the principal investigator to discuss the protocol and resolve queries prior to the full IRB meeting; (7) simultaneous submission of study documents to IRB, IDS, and PCLM; (8) express IRB approval was formulated to fast-track the approval process (within 15 days); and (9) development of standard operating procedure for the pathway of new industry-sponsored research. After that, a postassessment was done of five newly conducted sponsored CTs to evaluate the effect of the implemented interventions on streamlining and reducing the cycle time of the startup phase of new sponsored CTs.

### Ethical approval

The approval to conduct the study was obtained from the KFMC IRB.

### Statistical analysis

The mean time for various metrics of the CT startup was calculated before and after implementation of the intervention protocol to analyze and compare the percentage reduction in the mean cycle time for the newly sponsored clinical studies. The time required for the accomplishment of different tasks is represented as mean (SD), and statistical significance among groups was determined by one-way single-factor analysis of variance, with *P* < 0.05 considered as statistically significant.

## Results

**Fig. 1:**
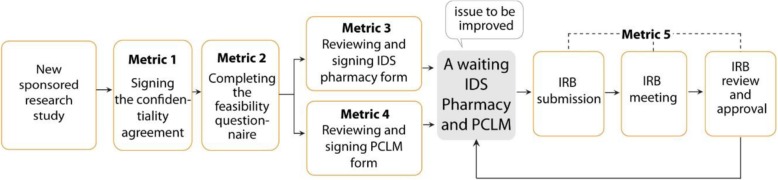
Process: the flow of the original startup phase

The original startup phase used to take 24.8 weeks, resulting in a severe delay. This occurred due to deferral in the PCLM and IDS pharmacy approvals (Fig. 1). Therefore, in the original startup phase, approvals from IDS pharmacy and PCLM must be in place before the study package is submitted to the IRB. This would help the trial to be on track and prevent any apparent scope of delay for subject recruitment.

**Fig. 2:**
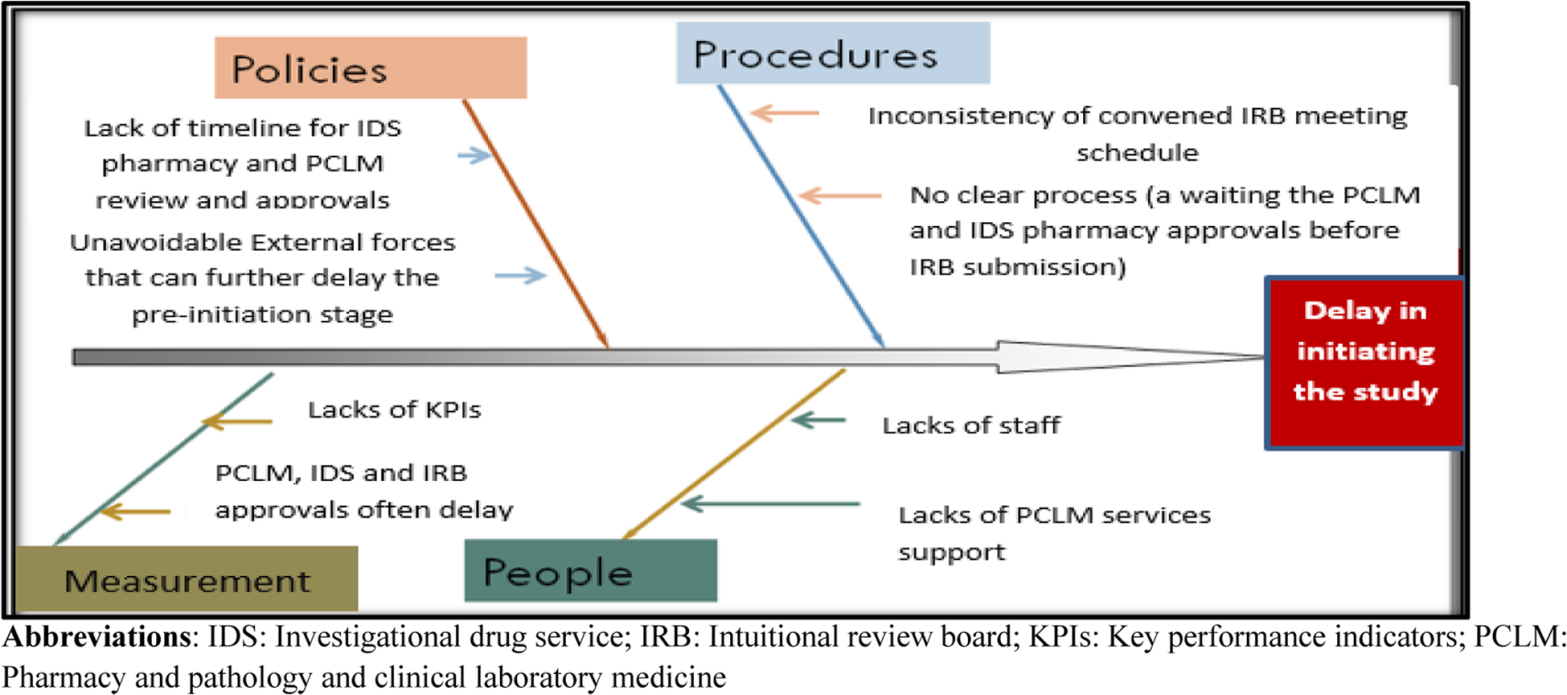
Fishbone analysis of the factors leading to clinical trial startup delay

Our fishbone diagram identified several factors in the delay in the current startup phase (Fig. 2). The analysis revealed lack of a well-defined timeline for IDS pharmacy and PCLM to review and approve the study protocol. Further, the absence of measurements by key performance indicators to monitor the performance, lack of staff, and finally the inconsistent convening of the IRB meetings were also considered to significantly affect the delay in the startup phase of the CT.

**Fig. 3:**
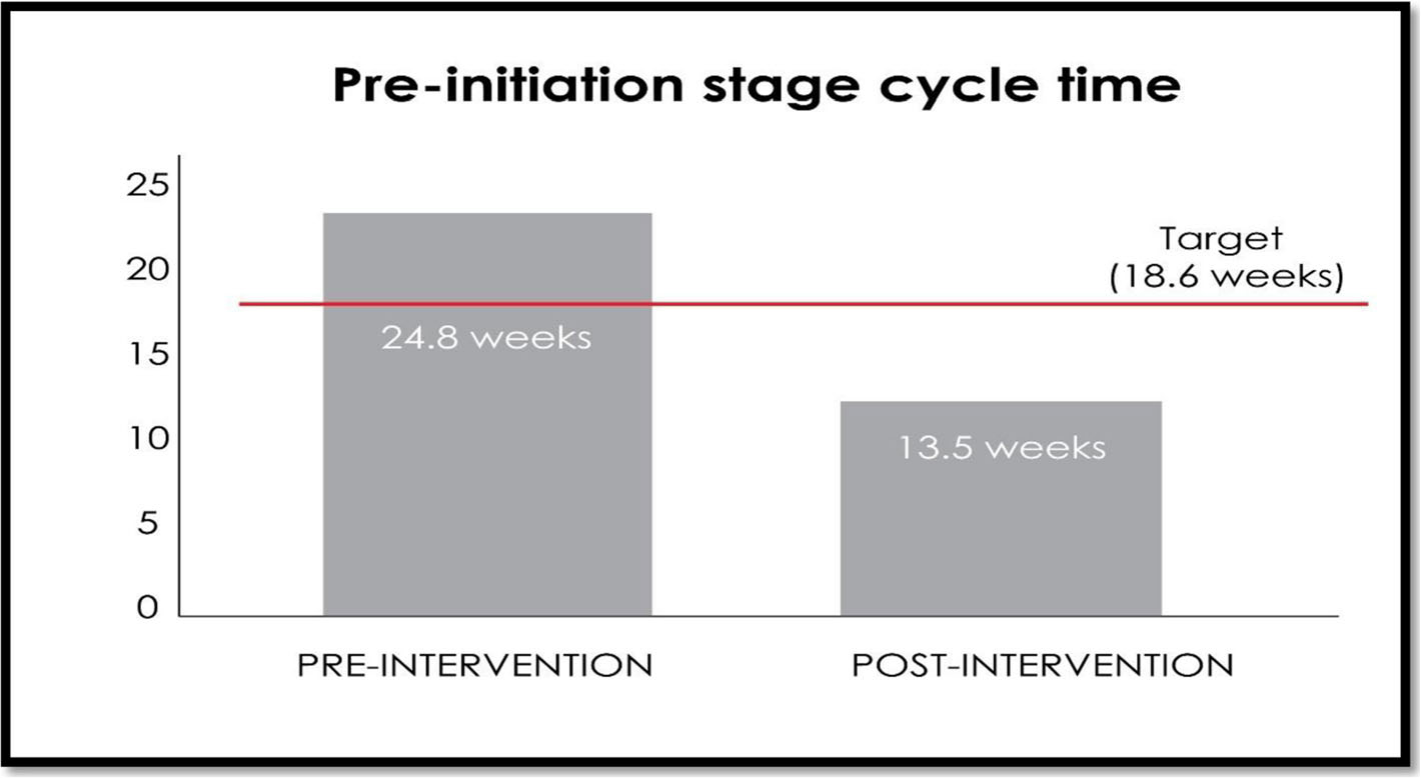
Cycle time of the preinitiation stage of the new sponsored clinical studies pre- and postintervention

Before the implementation of the new interventional steps, the mean cycle time of the startup phase of the new sponsored clinical studies used to be 24.8 weeks. After the intervention, the whole process was reduced to 13 weeks (Fig. 3, Table 1). Although 45.6% shortening of the entire CT life cycle was noted with the new interventional protocol, the change was not significant (*P* = 0.36) (Table 2). The mean [SD] time for the PCLM approval before the intervention (1.7 [2.0] weeks) also showed a 60.4% improvement after the implementation of the interventions (0.7 [0.94] weeks). However, the change was not significant (*P* = 0.59). IDS and IRB approval time mean (SD) also showed a marked improvement from 4.2 (3.6) and 7.5 (3.1) weeks to 1.5 (2.7) and 6.6 (5.7) weeks, respectively. Previously, the IRB meeting was organized in an approximately 45-day interval. After the implementation of the interventions, the frequency of IRB meetings showed an improvement, as shown in Table 1. But the improvement was not statistically significant (*P* = 0.27).

**Table 2:** Comparison of mean time for accomplishing different tasks for a clinical trial startup phase before and after implementation of the intervention protocol

	Before implementing the intervention	After implementing the intervention	*P* value	Percentage reduction
	Mean [SD] (week)	Mean [SD] (week)		
PCLM approval	1.7 [2.0]	0.7 [0.94]	0.59	60.4
IDS approval	4.2 [3.6]	1. 5 [2.7]	0.27	64.9
IRB approval	7.5 [3.1]	6.6 [5.7]	0.27	12
From confidentiality agreement to IRB approval	24.8 [8.2]	13.5 [11.6]	0.36	45.6

*Abbreviations: PCLM* pathology and clinical laboratory medicine, *IDS* investigational drug service, *IRB* institutional review board

## Discussion

The findings of our study demonstrated an overall reduction in the mean cycle time of the startup phase of newly sponsored CTs that gets prolonged unnecessarily due to ancillary services required at the beginning of the trial. Mainly, the inconsistent convening of the IRB meetings and the extended period needed to obtain approval from IDS pharmacy and PCLM on the study protocol were culprits in delaying the startup phase of a CT. Similar points were also noted by Giffin et al. [3]. According to Barbara Alving, Director, National Center for Research Resources, CTs experience a significant delay from the time of IRB submission to getting the ethical review complete. Alving further commented that it takes approximately 4–7 months to negotiate a clinical trial agreement between an academic institution and industry sources [3].

Although recent studies have reported improved trends in the overall conduct of CTs, sponsors continue to experience significant challenges in meeting overall CT timeline demands [13–16]. Sites must perform several specific activities related to documentation, submissions, agreement approval, and patient visit schedules [4–6]. According to Abozguia et al., in the United Kingdom, starting and conducting a clinical study is a complicated and time-consuming process with delay in getting the approval of funding, ethics committee approval, and approval for research and development from NHS R&D [4].

Krafcik et al. investigated the various timelines of finalizing the contract and budget, obtaining IRB approval, subject enrollment time, and total study startup time based on the study type. The time required for IRB approval was similar between study types (device, biologic, drug, observational) with 46.8 days average time [6]. In our studies, the time for the IRB approval was approximately 52.5 days. Historically, the study startup phase has been viewed as a labor-intensive, costly, and time-consuming component of the CT process. Various inefficiencies and limitations continue to threaten prompt study startup [6].

The study also reported a faster execution of the startup phase and better subject enrollment with more rapid IRB approval [6]. Further, a study conducted by Hurley et al. demonstrated that the CT activation period could be reduced through appropriate tools (web-based collaborative workflow tracking tool), staffing, leadership, and setting proper priorities. The trial activation times for the six studies used as tests of change were 49, 54, 78, 58, 62, and 32 days. The key activities included during the activation phase of the CTs were IRB preparation, Medicare coverage analysis processing, protocol review and monitoring committee review, Medical Research Council review, budget negotiation, and contract execution. However, a delay of more than 6 weeks observed was mainly due to sponsors [17].

The present study provides better insight and understanding of the various steps involved in the startup phase of a CT. These can efficiently help in reducing the time lag period in initial CT stages and its associated costs. However, large multicenter studies are required to further support the present findings.

The emergence of newer approaches and strategic intervention protocols to streamline burdensome and time-consuming preinitiation procedures offers promise; however, with the still uneven adoption of automated and integrated data systems, challenges in predicting startup timelines, and identifying potential holdups will continue. Elimination of the outsider forces to avoid further delay in the preinitiation stage is a prerequisite for the timely conduct of a CT.

### Study limitations

The number of studies pre- and postintervention were small. Hence, the results cannot be generalized. Furthermore, a detailed study considering a larger number of trials can provide better insight.

## Conclusion

Although improvement has been made in the way startup phase activities are conducted, there remains much work to be done if actual efficiencies are to be achieved in CT performance to increase predictability in site startup. Our study pointed out the root causes of startup delay and their proposed intervention to overcome the shortcomings. A detailed study considering a larger population of trials can provide more insight. Moreover, the timeline of various startup stages is expected to vary greatly among different countries. This is due to different infrastructure and regulations of various regulatory bodies that influence the modulation of the startup phase trial period. Research conducted at clinical research sites can be minimized if different departments and committees are willing to work together to recognize inefficiencies, set organizational priorities, and streamline processes.

## Data Availability

Available upon request.
